# Synchronized purification and immobilization of his-tagged β-glucosidase via Fe_3_O_4_/PMG core/shell magnetic nanoparticles

**DOI:** 10.1038/srep41741

**Published:** 2017-01-30

**Authors:** Yang Zhou, Shaofei Yuan, Qian Liu, Dandan Yan, Yun Wang, Li Gao, Juan Han, Haifeng Shi

**Affiliations:** 1 Institute of Life Sciences, Jiangsu University, 301# Xuefu Road, Zhenjiang 212013, China; 2 School of Chemistry and Chemical Engineering, Jiangsu University, 301# Xuefu Road, Zhenjiang 212013, China; 3 School of Food and Biological Engineering, Jiangsu University, 301# Xuefu Road, Zhenjiang 212013, China

## Abstract

In this paper, an efficient and convenient Fe_3_O_4_/PMG/IDA-Ni^2+^ nanoparticles that applied to purify and immobilize his-tagged β-glucosidase was synthesized, in which, Fe_3_O_4_/PMG (poly (*N, N’-*methylenebisacrylamide-co-glycidyl methacrylate) core/shell microspheres were synthesized firstly using distillation-precipitation polymerization, then iminodiacetic acid (IDA) was used to open epoxy rings on the shell of microspheres to the combination of Ni^2+^. The gene of β-glucosidase that was from *Coptotermes formosanus Shiraki* was amplified, cloned into the expression vector pET28a with an N-terminal His-tag, and expressed in *E.coli* BL21. The nanoparticles showed the same purification efficiency as commercial nickel column which was a frequently used method in the field of purifying his-tagged proteins from crude cell lysates. The results indicated that Fe_3_O_4_/PMG/IDA-Ni^2+^ nanoparticles can be considered as an excellent purification material. β-glucosidase was immobilized on the surface of Fe_3_O_4_/PMG/IDA-Ni^2+^ to form Fe_3_O_4_/PMG/IDA-β-glucosidase by means of covalent bound with imidazolyl and Ni^2+^. The immobilized β-glucosidase exhibited excellent catalytic activity and stabilities compared with free β-glucosidase. In addition, immobilized β-glucosidase can be recycled for many times and retain more than 65% of the original activity. The materials display enormous potential in the aspect of purifying and immobilizing enzyme.

*Coptotermes formosanus Shiraki* is a well-known wood-feeding termite that mainly feeds on plant cellulose and related products. It can degrade efficiently lignocellulose polysaccharides because of its unique multi-enzyme catalysis system in which β-glucosidase (BG) plays a vital role^1^. BG belongs to the family of glycoside hydrolase and widely exists in various kinds of microorganisms, such as bacteria, fungi. It involves in glucose metabolism and plays an important role to keep balanced biological functions for organisms. In addition, BG can hydrolyze flavor precursors from fruits, vegetables, tea to aroma substance possessed rich natural flavors^2^. Meanwhile, it can degrade cellulose to generate glucose, which can further be fermented to ethanol^3^. Currently, the method used to purify protein with his-tagged is nickel column purification. Although the method can gain more pure protein, it still has some disadvantages. Firstly, the price of nickel column is costly. Secondly, the nickel column needs to be regenerated after using about three times. Thirdly, the purity of his-tagged protein was low. Because of the above-mentioned disadvantages, a more effective method that can be used to purify his-tagged proteins is urgently needed. Meanwhile, the free enzymes have some weakness of large consumption, difficult separation and recycling process, hence increasing the utilization of enzymes is also a key in industrial production.

Recent years, the development of enzyme immobilization provides an efficient method for us to solve the problem. Enzyme immobilization has been widely studied owing to its advantages, such as enzyme reutilization, enhanced thermal stability and easy separation from mixture^4^. The methods of immobilization commonly included adsorption^5^,^6^, combination^7^, and entrapment^8^,^9^. With the increase in application demand of the enzyme, materials that used for immobilization also have been updated constantly. Among these materials, magnetite (Fe_3_O_4_) nanoparticles have been considered suitable for immobilization because of their multifunctional characteristics, including the small size, high surface area for the attachment of enzymes^10^, superparamagnetism and low toxicity. Most importantly, they can be easily separated from the reaction system. The process of separation only depends on the external magnetism, without centrifuges, filters or other expensive equipment^11^,^12^.

In this work, the magnetic core-shell structured Fe_3_O_4_/PMG/IDA-Ni^2+^ nanoparticles were prepared, in which the core consisted of a Fe_3_O_4_ nanoparticle, and the surface of the shell was covered with abundant Ni^2+^. Firstly, we separated BG by using these nanoparticles compared to nickel column. Then, BG was immobilized on their surface through combination with the imidazole group and Ni^2+^ to form Fe_3_O_4_/PMG/IDA-BG. The immobilization conditions, such as the added amount of BG, the incubating time and incubating temperature, were investigated. Subsequently, optimal pH, temperature, thermal and storage stabilities, kinetic parameters, and reusability assay were also studied.

## Results and Discussion

### Characterization of nanoparticles

**Figure 1:**
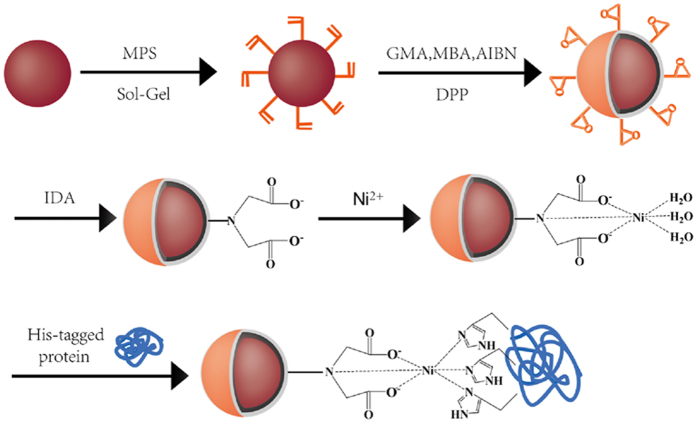
The diagram of the preparation of Fe_3_O_4_/PMG/IDA nanometer materials and its binding proteins.

**Figure 2:**
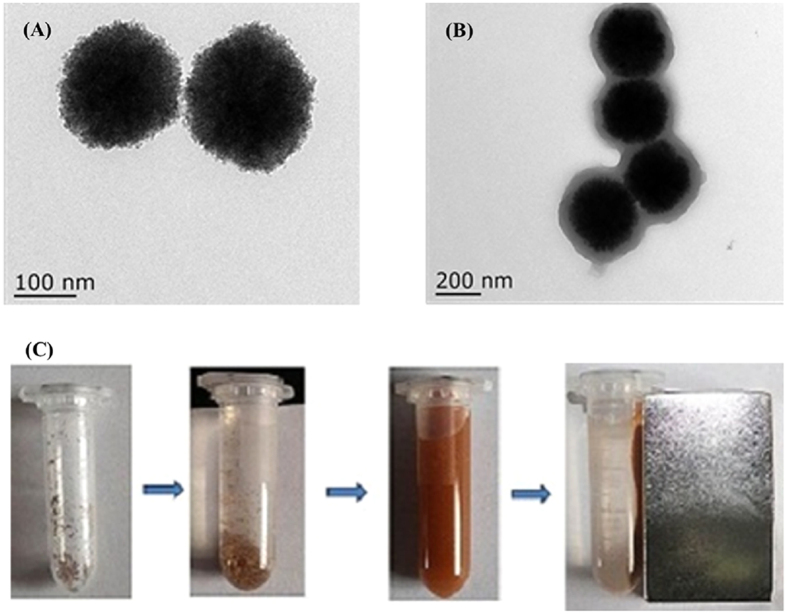
TEM images of **(A)** Fe_3_O_4_, **(B)** Fe_3_O_4_/PMG and **(C)** superior dispersibility.

According to the above-mentioned procedure, Fe_3_O_4_/PMG/IDA-Ni^2+^ nanoparticles were successfully prepared. Figure 1 showed the specific procedure of nanoparticles synthesis and combination between nanoparticles and his-tagged BG. Representative TEM images of the Fe_3_O_4_ and Fe_3_O_4_/PMG nanoparticles are shown in Fig. 2A,B. The Fe_3_O_4_ nanoparticles have an average diameter of about 200 nm (Fig. 2A), and were uniform both in shape and size. After being encapsulated with PMG, the size of the composite microspheres increased to about 350 nm (Fig. 2B). The obtained Fe_3_O_4_/PMG nanoparticles possessed a well-defined core shell structure and superior dispersibility in aqueous media (Fig. 2C).

**Figure 3:**
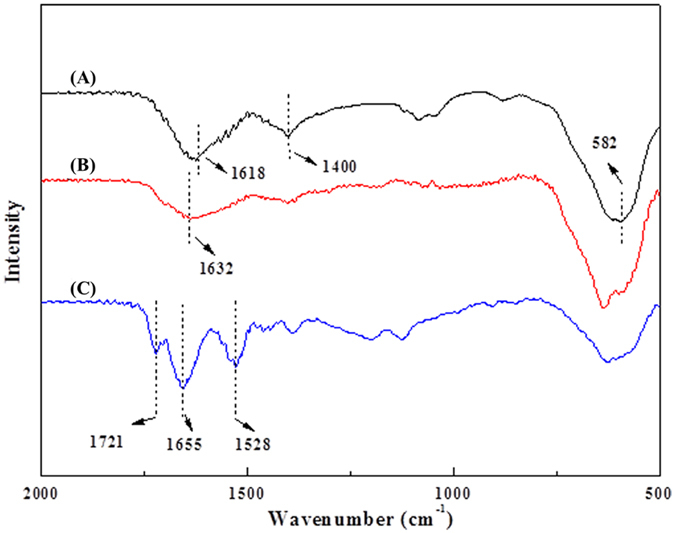
FT-IR spectra of **(A)** Fe_3_O_4_, **(B)** Fe_3_O_4_-MPS and **(C)** Fe_3_O_4_/PMG.

Figure 3 shows the FT-IR spectra of the naked and functionalized magnetic nanoparticles. As shown in Fig. 3A, the peak at 582 cm-1 was attributed to the Fe-O bond, and the peaks at about 1,618 cm^−1^ and 1,400 cm^−1^ were associated with carboxyl groups available from the stabilizer citrate. As shown in the FT-IR spectrum of the Fe_3_O_4_-MPS nanoparticles (Fig. 3B), the peak at 1,632 cm^−1^ corresponds to the stretching vibration of C = C bond of MPS. The successful functionalization of Fe_3_O_4_ with PMG (Fig. 3C) is demonstrated by the absorption peak of C = O at 1,721 cm^−1^ in GMA, and N-H at 1,528 cm^−1^ in MBA, respectively. These data further prove that the polymer-GMA is successfully grafted onto the Fe_3_O_4_ nanoparticles surface.

**Figure 4:**
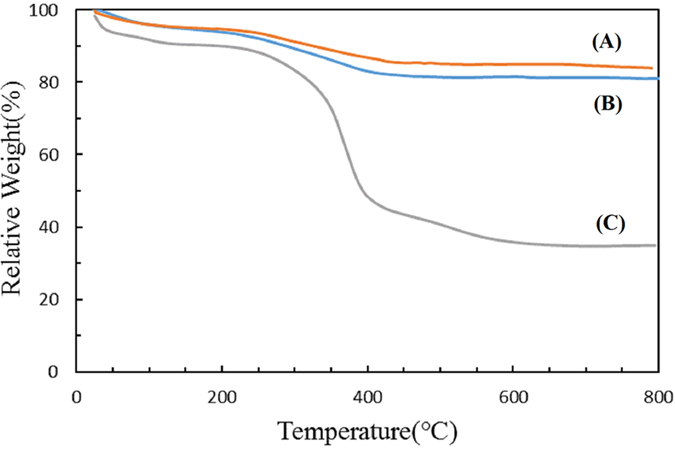
Thermogravimetric analysis of **(A)** Fe_3_O_4_, **(B)** Fe_3_O_4_-MPS and **(C)** Fe_3_O_4_/PMG.

Components of composite microspheres were measured by thermogravimetric analysis (TGA) (Fig. 4). While the organic components decomposited and inorganic components remained at high temperature. The 17.09 wt% loss of Fe_3_O_4_ is attributed to the weight ratio of citrate stabilizer and the physically adsorbed water. After modified by MPS, the loss of 19.76 wt% was assigned to the physically adsorbed water and small amount of MPS on the magnetic surface. When the outmost PMG layer was introduced, the weight loss of the composite microspheres was about 65.07 wt%, which is much higher than Fe_3_O_4_-MPS (19.76 wt%). The first weight loss (10.3%) until 200 °C was due to the evaporation of the physically adsorbed water or solvent, and the second major weight loss (54.77%) from 200 to 600 °C was due to the decomposition of the polymer component in the shell layer of the corresponding microspheres. And the magnetite content of Fe_3_O_4_/PMG is about 34.93 wt%.

**Figure 5:**
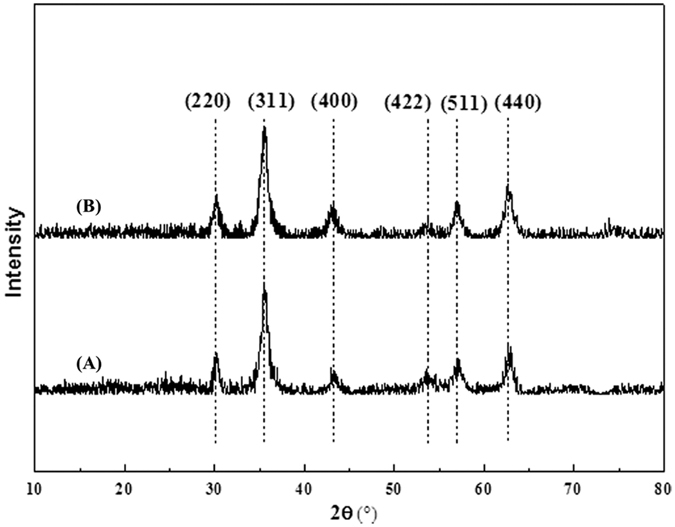
XRD patterns of **(A)** Fe_3_O_4_ and **(B)** Fe_3_O_4_/PMG.

The crystalline structure and phase purity of the Fe_3_O_4_ and Fe_3_O_4_/PMG nanoparticles were determined by powder XRD. As shown in Fig. 5, six characteristic peaks (2θ = 30.1°, 35.5°, 43.1°, 53.4°, 57.0° and 62.6°) were indexed as (220), (311), (400), (422), (511) and (440), respectively, which can be matched well with the standard XRD data of Fe_3_O_4_ (JCPDS 19-629). These results revealed that the crystal structure of the magnetic component was unchanged during the whole modification process.

**Figure 6:**
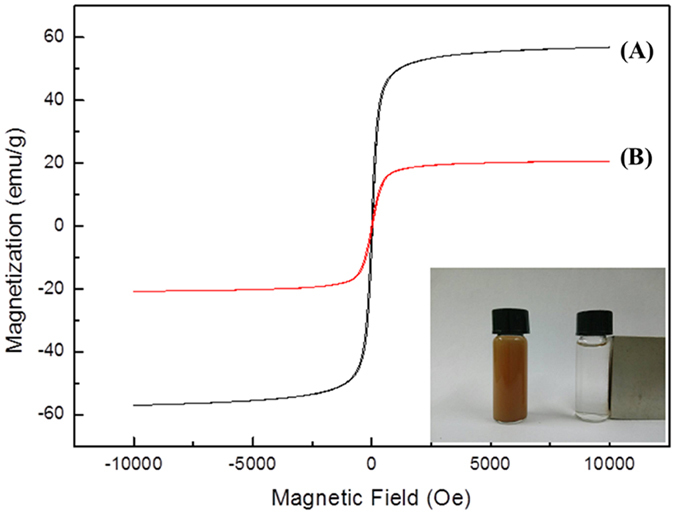
Magnetic hysteresis curves of **(A)** Fe_3_O_4_ and **(B)** Fe_3_O_4_/PMG. The insert was the phonomenon for Fe_3_O_4_/PMG separating from water in 30 s.

The magnetic properties of Fe_3_O_4_ and Fe_3_O_4_/PMG microspheres were studied by a vibrating sample magnetometer (VSM) at room temperature. The saturation magnetization values for Fe_3_O_4_ and Fe_3_O_4_/PMG microspheres were 56.9 and 20.7 emu/g as summarized in Fig. 6, respectively. The magnetic susceptibility of Fe_3_O_4_/PMG microspheres is large enough to be separated from the solution by quickly using a magnetic block.

### Application in protein purification

**Figure 7:**
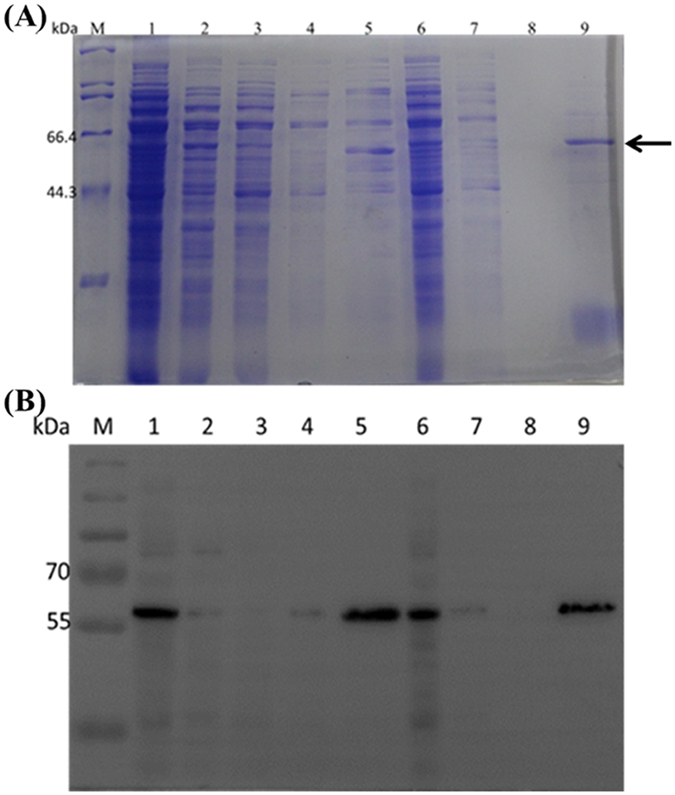
SDS-PAGE (**A**) and western blotting (**B**) analyses of BG isolated from crude *E.coli* lysate by Ni-charged resin (Lane 1–5) and Fe_3_O_4_/PMG/IDA nanoparticles (Lane 6–9). **(A)** M: protein molecular weight marker (Broad). **(B)** M: PageRuler Plus Prestained Protein Ladder; Lane 1 is crude cell lysate of BG; Lane 2 and 6 is surplus crude cell lysate of BG after incubating with Ni-charged resin and Fe_3_O_4_/PMG/IDA; Lane 3 and 7 is the flowing solution that Ni-charged resin and Fe_3_O_4_/PMG/IDA-BG were washed using solution I; Lane 4 and 8 is the flowing solution that Ni-charged resin and Fe_3_O_4_/PMG/IDA-BG were washed using solution II; Lane 5 and 9 is purified BG.

Following the above method, we tested the binding and separating ability of Fe_3_O_4_/PMG/IDA-Ni^2+^ with his-tagged BG, which has a molecular weight of 56 KDa. When it comes to separating ability, a comparison was done between Ni-charged resin and Fe_3_O_4_/PMG/IDA-Ni^2+^. Figure 7A showed that nearly 97% of his-tagged BG can be purified by Ni-charged resin and Fe_3_O_4_/PMG/IDA-Ni^2+^. What is more, we can see that the purity of β-glucosidase that was gained by nanoparticles is higher than that of Ni-charged resin from lane 5 and 9. Lane 5 (Fig. 7A) had other bands except for target band that was labeled by the black arrow. However, there was only the target band in lane 9 (Fig. 7A). Further, we used the technology of western blotting to verify the purification result. In the Fig. 7B lane 5 and 9 respectively displayed an apparent band that appeared using anti his-tagged antibody. Table 1 revealed the purification fold of BG from Ni-charged resin and Fe_3_O_4_/PMG/IDA-Ni^2+^. Nickel column purification is considered to be a high-efficiency method to purify his-tagged protein. In the Table 1, purification fold of nickel column and nanoparticles was 18.3 and 17.3, respectively. The value was approximately between the two. So to some extent, we can think that purification effect of nanoparticles is equivalent to that of nickel column. The nanoparticles will be able to be applied widely in the field of purification.

### Optimal conditions of immobilization

**Figure 8:**
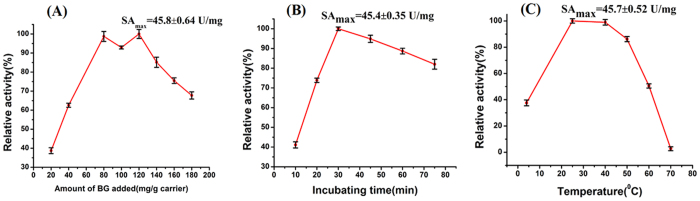
Optimal conditions of immobilization. **(A)** Amount of BG added. **(B)** Incubating time. **(C)** Temperature.

In the assay of testing optimal conditions, the following method was used with two quantitative factors and, one variable factor. Then, a certain amount of immobilized enzyme was obtained to detect enzyme activity. The relevant graphs are shown in Fig. 8(A,B and C). The maximum amount of BG with the incubating time and incubating temperature is 120 mg BG/g carriers, 30 min and 25 °C, respectively. It was found that, with the increment of variable, relative activity gradually raised and began to reduce or kept smooth when variable got to a certain point. One reason is that the increment of amount of enzyme leads to the reduction of combination opportunity between enzyme and nanoparticles. Each enzyme molecule competes to combine with nanoparticles, which gives rise to the partial combination between enzyme and nanoparticles within the incubation time. Another reason is that high temperature destroyed the structure of the enzyme, which causes his-tagged to be wrapped. Meanwhile, the destruction is irreversible.

At last, the immobilized BG were prepared at optimal amount of BG added (120 mg/g carriers), incubating time (30 min) and temperature (25 °C) for future study, supported by Fe_3_O_4_/PMG core/shell magnetic nanoparticles. Meanwhile, we calculated the binding capacity of Fe_3_O_4_/PMG/IDA-Ni^2+^ to BG in the optimal conditions of immobilization. After incubating 30 min, the supernatant was removed. The nanoparticles loaded BG were washed using Tris-HCl buffer (50 mM, pH8.0) for some times. According to the formula in the method, we gained the binding capacity of Fe_3_O_4_/PMG/IDA-Ni^2+^ to his-tagged BG is approximately 60 mg/g (BG/nanoparticles).

### Effects of pH and temperature on the enzyme activity

**Figure 9:**
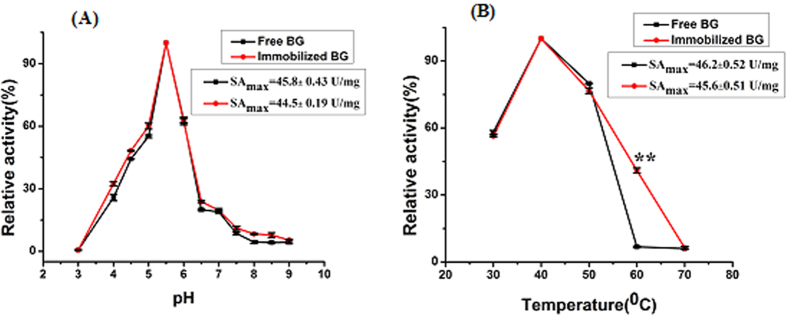
Effects of **(A)** pH and **(B)** temperature on the enzyme activity.

The effects of pH and temperature on the activities of immobilized BG were studied compared to free BG. Various pH in the reaction system could affect the activity of enzymes^13^. The effects of different pH values (3.0–9.0) on the activity of free and immobilized enzymes were compared at 40 °C, and the results were displayed in Fig. 9A. The curve shows that both enzymes have the same optimal pH at pH5.5.

The effect of temperature on the catalytic rate of enzymes mainly relied on its activity. With the increment of temperature, the heat motion of the enzyme and substrate also increase. Therefore, more collisions between the substrate and the enzyme’s active site occur, causing more enzyme–substrate complexes and finally more product compounds will be formed^14^. As shown in Fig. 9B, the optimal reactive temperature for free BG was similar to the immobilized BG that was 40 °C. When the temperature was higher than the optimal degree, the activities of both free and immobilized enzymes began to decrease. The immobilized BG exhibited relatively high temperature tolerance to retain 40% of its activity at 60 °C, while the free one only retained 7%.

### Immobilized stability

**Figure 10:**
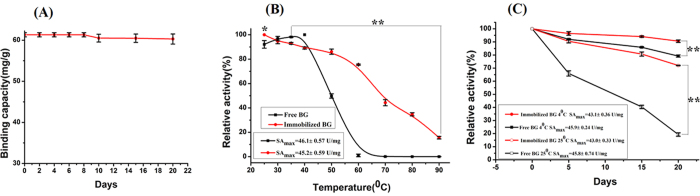
The experiment of stability. **(A)** Immobilization stability. **(B)** Thermal stability. **(C)** Storage stability dependent on time and temperature.

The stability of Fe_3_O_4_/PMG/IDA-BG was evaluated by incubating Fe_3_O_4_/PMG/IDA-BG in a Tris-HCl buffer (50 mM, pH8.0) at 4 °C, and monitored the amount of Immobilized BG on Fe_3_O_4_/PMG/IDA by detecting the concentration of supernatant after magnetic settlement. No substantial free BG from Fe_3_O_4_/PMG/IDA-BG was detected over a 20-day period of incubation. As Fig. 10A exhibited, Fe_3_O_4_/PMG/IDA-BG is sufficiently stable in the Tris-HCl buffer (50 mM, pH 8.0).

### Thermal stability

In general, every kind of enzyme has its own optimum temperature. The enzymatic activity is maximal at the optimum temperature, and under or exceed the temperature enzymatic activity reduces. In consequence, finding a method to raise thermo-stability of the enzyme is imminent in order to improve their catalytic activity. After heating for 30 min, the activities of the same quality of free and immobilized enzyme were evaluated according to 2.5. As the Fig. 10B described, free BG almost lost all activity when processed temperature reached 60 °C, nevertheless, that of immobilized BG still retained 75% of its initial activity. The loss of enzyme activity could be ascribed by the change of protein structure because of heating. While the binding of Fe_3_O_4_/PMG/IDA-Ni^2+^ and his-tagged BG avoid heat damage to some extent, accordingly active site of the enzyme is protected, which brought down the harm of high temperature. The similar phenomena had been discovered. For example, Gupta *et al*. prepared Cu-IDA and Cu-IDA-Sepharose that was used to immobilize bromelain^15^. Yang *et al*. applied Ni^2+^-PD-MNPs to immobilize his-tagged red fluorescent protein^16^. In their experiments, immobilized enzyme showed excellent thermal stability at high temperature (from 50 to 80 °C) compared to the free enzyme.

### Storage stability

Two copies of free and immobilized BG were prepared in advance. One copy of that stored in 4 °C, and another copy stored in 25 °C. The equal quality of both enzymes was used for the detection of enzymatic activity every four days until to 20th days. The results were depicted in Fig. 10C.

The storage stability of the immobilized BG was apparently higher than that of the free. Both BGs storing in 4 °C maintained the higher activities than those in 25 °C. The immobilized BG stored at 4 °C kept 91% of its original activity, and the free enzyme retained 80% of its activity at 4 °C. Free BG lost 80% of its primal activity at 25 °C at 20 days, the immobilized BG lost only about 25% of its activity. It suggests that the magnetism of Fe_3_O_4_/PMG/IDA-Ni^2+^ nanoparticles could hold BG in a stable state in comparison to the free enzyme.

### Reusability assay

**Figure 11:**
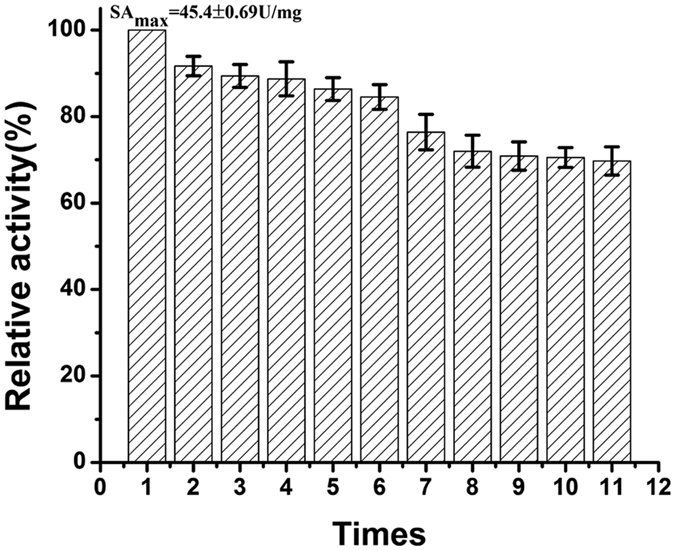
Reusability assay of Fe_3_O_4_/PMG/IDA-BG for catalyzing p-NPG.

Reusability of immobilized enzymes is a significant parameter when it comes to the significance of immobilization. Reusability of immobilized BG in this research was evidenced by its surplus activities at each round iteration. In every round, immobilized BG was incubated with the 4 mM p-NPG for 10 min, and then the reaction was ceased using 1 M sodium carbonate. Fe_3_O_4_/PMG/IDA-BG was separated by an external magnet and washed several times with Tris–HCl buffer (50 mM, pH8.0). Immediately following, sedimentary immobilized BG was suspended by Tris–HCl buffer (50 mM, pH8.0) and entered into the next round of usage. The cycle batch of immobilized BG is eleven times, and its activity retained approximately 70% as illustrated in Fig. 11. The excellent reusability could significantly reduce the operation cost in practical applications^17^. It could be explained by that immobilization of BG limited its freedom to resist conformational changes, and hence led to increasing stability toward denaturation.

In consideration of some excellent properties of immobilized BG in this research, a comparison was carried out between our immobilized enzyme and other magnetic immobilized enzymes that were reported in literature. There mainly included four aspects: magnetic saturation (Ms), binding capacity, thermal stability and retained activity of the reusage. The corresponding data was showed in the Table 2. These magnetic nanoparticles exhibited different advantages. Our magnetic nanoparticles are prior to others in the aspects of binding capacity and reusability by the comparison.

### Kinetic parameters

p-NPG was used as substrate in the BG activity assays. Kinetic parameters, the Michaelis constant (*K*_m_), the maximum rate of the reaction (*V*_max_) and the catalytic constant (*K*_cat_) for free and immobilized BG were measured using p-NPG at 2–16 mM concentrations. *K*_m_ and *V*_max_ were calculated from the Line weaver–Burk plots using the initial rate of the reaction data. From Table 3, we gained the affinity of immobilized BG to substrates is higher than that of free one, but for catalytic activity, the result was just the opposite.


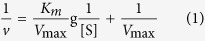


where [S] is the concentration of the substrate, *v* and *V*_max_ represent the initial and the maximal rate of the reaction, respectively. *K*_m_ is defined as the substrate concentration when reaction speed is equal to one half of the maximum reaction rate. *K*_m_ can reflect the affinity of the enzyme and substrate. The lower *K*_m_ is, the greater the affinity is. *K*_cat_ stands for catalytic constant. The bigger *K*_cat_ is the catalytic activity for substrates is better.

## Conclusion

In summary, a magnetic core-shell nanostructure, in which Fe_3_O_4_ magnetite is the core and PMG is the shell layer, has been synthesized by distillation precipitation polymerization. Fe_3_O_4_/PMG/IDA-Ni^2+^ nanoparticles exhibited better performance in the separation of His-tagged BG than that of nickel column purification. Especially, the binding capacity can reach to 60 mg/g. Compared with free BG, the immobilized BG showed stronger temperature resistances, better repeatability and stability. It indicates that the immobilized BG on the Fe_3_O_4_/PMG/IDA-Ni^2+^ nanoparticles have a potential application in the field of catalyst, such as improving flavor juice, degrading cellulose and defensing pest.

## Methods

### Materials

Iron(III) chloride hexahydrate (FeCl_3_·6H_2_O), trisodium citrate dehydrate (Na_3_C_6_H_5_O_7_·2H_2_O), ammonium acetate (NH_4_Ac), ethylene glycol (EG), anhydrous ethanol, acetonitrile, acetone, Iminodiacetic acid (IDA), sodium hydroxide (NaOH), and nickel(II) chloride hexahydrate (NiCl_2_·6H_2_O) were purchased from Sinopharm Chemical Reagents Company. Aqueous ammonia solution (25%) and γ-Methacryloxypropyltrimethoxy-silane (MPS) were purchased from Aladdin. Glycidyl methacrylate (GMA) was obtained from Aladdin and vacuum distilled. N, N′-Methylenebisacrylamide (MBA) was bought from Aladdin and recrystallized from acetone. 2, 2′-Azobis (2-methylpropionitrile) (AIBN) was purchased from Aladdin and recrystallized from ethanol. Deionized water was used in all the experiments. Glycine, Tris, SDS, Bromophenol blue, coomassie brilliant blueR250 and 4-Nitrophenol (p-NP) were purchased from Sangon Biotech (Shanghai, China). Acrylamide and TEMED were bought from Sigma (USA). IPTG and protein markers were purchased from TaKaRa (Dalian, China). p-NP-β-D-glucopyranoside (p-NPG) was purchased from BioTeke Corporation (Beijing, China). Purified BG was produced using a High Affinity Ni-NTA Resin (GenScript, Nanjing, China) according to the manufacturer’s instruction. Both of specific process can refer to Feng^1^ Protein concentrations were detected using Pierce^®^ BCA Protein Assay Kit (Thermo Scientific, USA).

### Preparation of Fe_3_O_4_/PMG/IDA−Ni^2+^ Nanoparticles

#### Synthesis of Fe_3_O_4_ Particles

The Fe_3_O_4_ particles with a core size around 200 nm were synthesized through a modified solvothermal reaction. Typically, FeCl_3_·6H_2_O (1.350 g), NH_4_Ac (3.854 g) and trisodium citrate dihydrate (0.400 g) were dissolved in ethylene glycol (70 mL). The mixture was stirred vigorously for 1 h at 160 °C to form a homogeneous black solution, and then transferred into a Teflon-lined stainless-steel autoclave (100 mL capacity). The autoclave was heated at 200 °C and maintained for 16 h, and then it was cooled to room temperature. The black product was washed with ethanol and separated from the solvent by using a magnet. The cycle of washing and magnetic separation was repeated for several times. The final product was dispersed in ethanol for further use.

#### Modification of the Fe_3_O_4_ with MPS

The modification of Fe_3_O_4_ with MPS was achieved by adding 40 mL of ethanol, 10 mL of deionized water, 1.5 mL of NH_3_·H_2_O, and 0.6 g of MPS into the Fe_3_O_4_ particles ethanol suspension and vigorously stirring the mixture for 24 h at 70 °C. The obtained product was separated by using a magnet and washed with ethanol to remove excess MPS. The resultant Fe_3_O_4_-MPS nanoparticles were dried in a vacuum oven at 40 °C until they reached a constant weight.

#### Synthesis of Fe_3_O_4_/PMG Core/Shell Nanoparticles

Coating a PMG layer onto Fe_3_O_4_-MPS nanoparticles was executed by the distillation-precipitation polymerization (DPP) of GMA, with MBA as the cross-linker and AIBN as the initiator, in acetonitrile. Typically, Fe_3_O_4_-MPS seed nanoparticles (50 mg) were dispersed in acetonitrile (40 mL) in a dried single-necked flask (100 mL capacity) under ultrasonic condition for 3 min. Then a mixture of GMA (150 mg), MBA (150 mg), and AIBN (6 mg) was added to the flask to initiate the polymerization. The flask, submerged in a heating oil bath, was attached to a fractionating column, Liebig condenser, and a receiver. The reaction mixture was heated from room temperature to the boiling state within 30 min and the reaction was ended after acetonitrile (20 mL) was distilled from the reaction mixture within 1 h. The obtained Fe_3_O_4_/PMG nanoparticles were collected by magnetic separation and washed with ethanol and water repeatedly.

#### Preparation of Fe_3_O_4_/PMG/IDA-Ni2^+^ Nanoparticles

IDA (0.33 g) and NaOH (0.2 g) were dissolved in 20 mL of deionized water under stirring. 2 M NaOH solutions were used to adjust the pH of the solution. Then Fe_3_O_4_/PMG nanoparticles (50 mg) were added to the solution, and the mixture was stirred vigorously for 12 h at 80 °C. The obtained Fe_3_O_4_/PMG/IDA nanoparticles were separated by a magnet and washed by ethanol and water for several times. Subsequently, 50 mg of Fe_3_O_4_/PMG/IDA nanoparticles was added to a 10 mL of 0.1 M NiCl_2_ solution and stirred for 2 h at room temperature. The product was separated by a magnet from the solution and washed several times with water. The resultant Fe_3_O_4_/PMG/IDA-Ni^2+^ nanoparticles were dried in a vacuum oven at 40 °C.

### Characterization

Transmission electron microscopy (TEM; Tecnai G2 F30 S-TWIN, USA) analyses were performed with an acceleration voltage of 300 kV. The crystal structure of the nanoparticles was detected using X-ray diffraction (XRD; Shimadzu, XRD-6100, Japan). The Fourier transform infrared (FTIR) spectroscopy analysis was conducted on a FTIR spectrometer (Nicolet Nexus 470, USA) in the range of 400–4,000 cm^−1^. Thermogravimetric analysis (TGA) was performed for power samples with a heating rate of 10 °C/min using a thermogravimetric analyzer (Netzsch STA 499 C, Germany) under nitrogen atmosphere up to 800 °C. The magnetic properties of the prepared nanoparticles were measured on a vibrating samples magnetometer (VSM, LakeShore 7410, USA).

### Construction of Recombinant Expression Plasmids and Expression of His-tagged BG

The coding sequences of BG (no. GQ911585) genes were searched from NCBI-GenBank and used to design primers (Table 4)^1^. The signal peptide and restriction enzyme cutting sites were analyzed by SignalP 3.0 Server and Webcutter 2.0, respectively. cDNA of *Coptotermes formosanus Shitake* was served as template to amplify the gene of BG by PCR technology. Following this, the PCR product was digested using two kinds of restriction endonuclease- Hind Ш and Xho Ι. Finally, the digested product was cloned to expression vector pET28a between the Hind Ш and Xho Ι restriction sites. The recombinant plasmid was verified by DNA sequencing.

The empty plasmid (only pET28a) and a recombinant plasmid expressing BG were transformed into BL21 (DE3). A signal colony was incubated in LB media with kanamycin by shaking at 37 °C overnight. Enlarged culture was done according to 1% between bacteria and LB media by shaking at 37 °C until OD_600_ = 0.4, at which time the temperature of shaker was lowered to 25 °C, isopropyl-β-D- thiogalatopyranoside (IPTG) was added to a final concentration of 0.2 mM. After shaking about 6 h, the cells were harvested by centrifugation at 4,500 g and 4 °C and stored at −80 °C.

The frozen cell was resuspended in Tris-HCl (50 mM, pH8.0) after being washed using Tris-HCl (50 mM, pH8.0) for twice, and then treated 30 min with lysozyme whose final concentration is 1 mg/mL on ice. At last, the suspension was broken by Ultrasonic Cell Disruptor at 30 min, in which precipitation and supernatant were separated by centrifugation at 12,000 rpm and 4 °C for 20 min twice and stored at −80 °C.

### Loading amount of Fe_3_O_4_/PMG/IDA to his-tagged BG

A certain volume of the protein solution was mixed with nanoparticles and incubated at 25 °C for 30 min. Subsequently, the supernatant was removed, and nanoparticles binding protein were washed with Tris-HCl buffer (50 mM, pH8.0) for some times until the concentration of the supernatant is zero. The amount of immobilized BG was determined by subtracting the amount of BG remaining in the Tris-HCl buffer from the BG added to immobilization. The BG loading amount was calculated from the following equation:


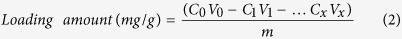


where *C*_0,1…x_ is the protein concentration and *V*_0,1…x_ is the volume of the free BG solution added to immobilization, respectively. *m* is the amount of nanoparticles.

### His-tagged BG separation from crude cell lysates using Fe_3_O_4_/PMG/IDA-Ni^2+^ and nickel column

Purification procedure using Ni-charged resin was according to standard protocol. At first, the column was loaded with 3–4 mL of Ni-charged resin, and the column was washed with 8 × bed volumes of equilibration buffer. Then, the crude cell lysates was incubated with Ni-charged resin at 4 °C. After 1.5 h, surplus cell lysates flowed out and were harvested. Ni-charged resin was washed with 3 mL solution I (50 mM Tris, pH8.0, 300 mM NaCl, 50 mM imidazole), 3 mL solution II (50 mM Tris, pH8.0, 300 mM NaCl, 100 mM imidazole), respectively. Finally, 2 mL solution III (50 mM Tris, pH8.0, 300 mM NaCl, 400 mM imidazole) was used to elute his-tagged BG from Ni-charged resin.

Fe_3_O_4_/PMG/IDA-Ni^2+^ nanoparticles suspension (200 μL, 2 mg/mL) were precipitated on a magnet and washed with 200 μL Tris-HCl (50 mM, pH8.0) for twice. Then, protein solution (600 μL, 2.632 mg/mL) was mixed with nanoparticles and incubated at 4 °C for 30 min. Subsequently, the supernatant was removed using Eppendorf, and nanoparticles binding protein were washed with 200 μL solution I (50 mM Tris, pH8.0, 300 mM NaCl, 50 mM imidazole), 200 μL solution II (50 mM Tris, pH8.0, 300 mM NaCl, 100 mM imidazole), respectively. At last, his-tagged BG was eluted from the nanoparticles with 200 μL solution III (150 mM Tris, pH8.0, 300 mM NaCl, 400 mM imidazole). The protein solutions from each step were analyzed by SDS-PAGE.

### Western blotting

Protein samples from separation of nickel column and nanoparticles were separated by SDS-PAGE and transferred to PVDF membrane. The membrane was subsequently blocked and incubated with primary antibody at 4 °C overnight. Anti-His-tag (1:2,000) was purchased from Abmart (Shanghai, China). The secondary antibody, anti-mouse (1:5000) was from Zhongshan Golden Bridge Biotechnology (Beijing, China). The blots were exposed to ECL Western Blotting Substrate (Vazyme, Nanjing, China).

### BG immobilization

Initially, three main factors were considered, including the amount of BG added, temperature and time. Fe_3_O_4_/PMG/IDA-Ni^2+^ nanoparticles (2 mg/mL) were dispersed in 200 μL of Tris-HCl (50 mM, pH8.0). Then, various volumes of the BG solution (20–180 μg BG) were added into suspension and the mixture was shaken at different temperatures (4–70 °C) for 10–75 min. The amount of BG immobilized on Fe_3_O_4_/PMG/IDA-Ni^2+^ was calculated by measuring the initial and final concentration of free BG in the solution using the Pierce^®^ BCA Protein Assay Kit.

### Assay of β-glucosidase activity

The enzymatic assays of p-NPG were performed as follows: the certain amounts of free and immobilized BG were incubated 10 min with 4 mM p-NPG substrates, 50 mM NaAc-HAc buffer at 40 °C. Assays were stopped by the addition of 1 M sodium carbonate. The measurement of color change was performed at 410 nm using spectrophotometer. The enzymatic assays were done three times, respectively. The final concentration of p-NP and corresponding value of OD_410_ were used for the standard curve construction^18^.

The specific activities (U/mg) of both free and immobilized BG were calculated with the following formula:


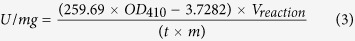


where *V*_reaction_ is the total volume of reaction; *t* is the reaction time; *m* is the mass of free and immobilized BG. The enzymatic activity assays results were calculated in accordance with 1 mg enzyme and the quantity of product per unit time.

## Additional Information

**How to cite this article**: Zhou, Y. *et al*. Synchronized purification and immobilization of his-tagged β-glucosidase via Fe_3_O_4_/PMG core/shell magnetic nanoparticles. *Sci. Rep.* **7**, 41741; doi: 10.1038/srep41741 (2017).

**Publisher's note:** Springer Nature remains neutral with regard to jurisdictional claims in published maps and institutional affiliations.

## Figures and Tables

**Table 1:** A Comparison of purification efficiency between magnetic nanoparticles and nickel column.

Proteins	Total protein (mg)	Specific activity (unit/mg)	Total activity (unit)	Yield (%)	Purification (fold)
Cell lysate ^a^(All proteins of *E.coli contained EP*)	8.2	0.064	0.5248		
Cell lysate with expression of BG ^b^(All proteins of *E.coli contained RP*)	9.7	2.53	24.541		
BG in cell lysate	9.7	2.466	23.92	100.0	1.0
Purified BG^c^	0.41	45.09	18.49	77.3	18.3
Purified BG^d^	0.34	42.61	14.49	60.6	17.3

^a,b^2.5 mL supernatant was obtained through ultrasonication after 100 mL *E.coli* was collected. ^c,d^2 mL purified BG was gained through nickel column chromatography and Fe3O4/PMG/IDA, respectively. EP: empty plasmid (pET28a). RP: the plasmid (pET28a) that was inserted into BG gene.

**Table 2:** Comparison of our immobilized enzyme and other magnetic immobilized enzymes that were reported in literature.

Magnetic nanoparticles	Enzyme immobilized	Ms (emu/g)	B.C (mg/g)	Thermostability	residual activity (%)
Fe_3_O_4_–APTES^19^	aminoacylase	n/a	n/a	47%/60 °C/1 h	40%/6 batches
chitosan/Fe3O_4_^20^	pullulanase	60.8	62.8	84%/60 °C/5 h	55%/8 batches
Fe_3_O_4_@MOF^21^	lipase	49.67	37.57	65%/65 °C/6 h	60%/10 batches
MNPs^22^	feruloyls esterase	n/a	n/a	50%/60 °C/72 h	35%/7 batches
chitosan coated γ-Fe_2_O_3_^23^	invertase	42	n/a	n/a	65%/20 batches
Fe_3_O_4_-CS^24^	β-Fructofuranosidase	n/a	n/a	n/a	55%/10 batches
Fe_3_O_4_/PMG/IDA-Ni^2+^	β-glucosidase	20.7	60	75%/60 °C/0.5 h	70%/11 batches

Ms: saturation magnetization. B.C: Binding Capacity. n/a: Not available or not possible to determine from the information published.

**Table 3:** Kinetic parameters of free and immobilized BG in hydrolysis of p-NPG.

Enzyme	*K*_m_ (mM)	*K*_cat_ (S^−1^)	*K*_cat_/*K*_m_ (S^−1^mM^−1^)
Free BG	4.2 ± 0.3	58.8 ± 1.2	14.0 ± 0.7
Immobilized BG	3.4 ± 0.4	45.9 ± 0.7	13.5 ± 0.7

**Table 4:** Oligonucleotide primers.

Target	Primer	Sequence
BG	PF	CCCAAGCTTGCATGGATGACGTCGATAACGAC(Hind Ш)
	PR	CCGCTCGAGTTAGTCTCGGAAGCGCTC(Xho Ι)
